# Factors Associated With Contraceptive Initiation and Use Among Women Who Have Given Birth in the Last Year: Findings From the 2023 Women's Reproductive Health Survey

**DOI:** 10.1111/1471-0528.18264

**Published:** 2025-07-09

**Authors:** Catherine Stewart, Amy Hough, Melissa J. Palmer, Ona McCarthy, Rebecca S. French, Neha Pathak

**Affiliations:** ^1^ Reproductive Health Research Department UCL EGA Institute for Women's Health London UK; ^2^ Institute of Global Health University College London London UK; ^3^ University Hospitals Birmingham NHS Trust Birmingham UK; ^4^ Faculty of Public Health and Policy The London School of Hygiene and Tropical Medicine London UK; ^5^ Mortimer Market Centre, Central North West London NHS Foundation Trust London UK; ^6^ University College Hospital, University College Hospitals NHS Foundation Trust London UK

**Keywords:** contraception, contraception initiation, contraception use, contraceptive counselling, fertility intentions, post‐natal contraception

## Abstract

**Objective:**

This study investigates contraceptive use among individuals who have recently given birth. It aims to identify factors associated with contraceptive use at two months and one‐year of giving birth. Additionally, it assesses whether receiving and being satisfied with contraceptive counselling influences contraceptive use.

**Design:**

Cross‐sectional analysis of data from the 2023 Women's Reproductive Health Survey.

**Setting:**

England.

**Population:**

Participants who had given birth in the last year (*n* = 2044).

**Methods:**

Bivariate and multivariable logistic regressions examined factors associated with contraceptive use.

**Main Outcome Measures:**

Contraceptive initiation within two months of giving birth. Contraceptive use within one year of giving birth.

**Results:**

Overall, 73% (*n* = 1489/2044) of participants were using contraception, however 58% (*n* = 1154/1998) had not initiated use within two months of giving birth. Bivariate analyses indicated that younger participants, those who received counselling, and those satisfied with it had higher odds of initiating contraception within two months. Similarly, younger individuals, those not desiring more children, and those who received counselling had higher odds of using contraception within one year. Multivariable analysis showed that receiving counselling and being satisfied with it were significantly associated with early initiation.

**Conclusions:**

This study highlights the need for high‐quality contraceptive counselling as part of maternity care. Counselling during or immediately after pregnancy was shown to impact contraceptive initiation and use. Policy improvements and further research on achieving universal high‐quality counselling are important to ensure equitable access to contraception.

## 1 Introduction

Choosing if and when to have children, and spacing births appropriately to fertility intentions are key principles of reproductive autonomy [1]. Knowledge, choice and timely access to effective and safe methods of contraception are essential to enable these decisions.

The time period after giving birth is a key time in an individual's reproductive life and women and birthing people are able to get pregnant again very quickly, as soon as 21 days after birth [2]. Therefore, access to and use of effective contraception within this period is crucial. However, little is known about preferences and use of methods after birth in the UK.

For those who would like more children, an interpregnancy interval of at least 18 months is recommended to prevent complications arising from short interpregnancy intervals such as preterm birth and adverse neonatal outcomes [3, 4]. Despite this recommendation, knowledge around interpregnancy intervals among women and birthing people who have recently delivered is limited [5]. In the UK demand for abortion services is increasing, and studies have shown that 1 in 13 individuals requesting an abortion have given birth in the last year [6, 7], indicating an increasing unmet need for contraception among this group.

Around half of women and birthing people will have resumed sexual activity within eight weeks of giving birth [8, 9]. Consequently, contraceptive counselling and provision at the six‐week postnatal GP check could be too late, and it may not be possible to provide effectively given the time constraints [10]. National Institute for Health and Care Excellence (NICE) and Faculty of Sexual & Reproductive Health (FSRH) guidance recommends that all methods of contraception should be available in maternity settings prior to discharge [2, 11]. Studies have also shown that this would be a preferred setting to receive contraception after birth [12, 13]. Despite this, data relating to the use of contraception in the early postnatal period, including timing of initiation and method choice, is limited.

This paper aims to investigate contraceptive use among women and birthing people who have given birth in the last year in England. The three main objectives are:

1. Identify the factors associated with contraceptive initiation within two months of giving birth and contraceptive use within one year.
2. Assess whether there is an independent association between receipt of and satisfaction with contraceptive counselling during or immediately after pregnancy and contraceptive initiation within two months or contraceptive use within one year.
3. Investigate the different contraceptive methods used within one year of giving birth.

## 2 Methods

### 2.1 Study Setting and Design

The 2023 Women's Reproductive Health Survey for England (RHSE) was conducted by researchers at The London School of Hygiene and Tropical Medicine and was funded by the Department of Health and Social Care [14, 15]. Data were collected between 6th September and 19th October 2023, from 59 332 people (52 129 completed the survey in full) who were assigned female at birth, aged 16–55 years and living in England. Participants were primarily recruited via social media (paid‐for advertising on Facebook and Instagram), news coverage of the survey in the UK media, and network dissemination through four UK‐based organisations (LGBT Foundation, Race Equality Foundation, Brook, and Birth Companions). Potential participants were directed to an online information sheet and consent form and asked to complete the questionnaire via snapsurveys.co.uk [16]. The questionnaire included a range of topics, including menstruation, menopause, contraception, pregnancy, obstetric experiences and gynaecological conditions.

### 2.2 Participants

All respondents were asked if they had ever been pregnant. If yes, they were asked if they had ever given birth, and if yes again, they were asked if they had given birth to a live baby in the last year. Questions were not mandatory and could be skipped, there was also routing within the questionnaire to ensure respondents were only asked questions that were relevant to them. Participants were included in this analysis if they answered yes to having a live birth within one year of completing the 2023 RHS (*n* = 2073).

### 2.3 Variables

Factors hypothesised to be associated with contraceptive initiation within two months of giving birth and contraceptive use within one year of giving birth were investigated. The factors investigated were: age, marital status, education, employment, deprivation, financial status, ethnicity, religion, migration status, parity, whether they had received contraception advice during or immediately after pregnancy and whether they were satisfied with the contraception advice they received during or immediately after pregnancy. For contraceptive use within one year, we also investigated fertility intention and sexual activity in the past six months. These variables were selected based on the published literature and the data available, demonstrating an association with postpartum contraceptive initiation [17].

Contraceptive initiation is a binary variable (y/n) of whether a woman had initiated contraception within 2 months of giving birth or not. This variable was created from the answer to the question ‘Did you start using a method of contraception within two months of this pregnancy ending?’. Contraceptive use is also a binary variable (*y*/*n*) of whether a woman was using a method of contraception at the time of the survey (within one year of giving birth) or not. This variable was created from the answer to the question ‘What contraceptive method are you currently using?’. Respondents were presented with a list of options, if they selected any method of contraception, we categorised them as using contraception, if they selected ‘no method used at the moment’ we classified them as not using contraception. For contraceptive effectiveness we constructed a new ordered categorical variable from the contraceptive type data to represent four categories of contraceptive effectiveness; no method, least effective methods (which includes condoms, cap/diaphragm, lactational amenorrhoea, fertility awareness and withdrawal), effective methods (which includes contraceptive pills, injection and patch) and then most effective methods (which includes hormonal and non‐hormonal IUDs, implant and sterilisation).

Fertility intention is an ordered categorical variable based on the answer to a multiple‐choice question which asked participants which statement best described the way they feel about having more children. The answer options were (1) do not want any more children; (2) not sure if want any more children; (3) definitely want more children—not currently trying; (4) currently trying. This variable is fertility intention at the time of the survey.

Sexual activity is a binary variable (*y*/*n*) of whether a woman had vaginal intercourse within the last 6 months (at the time of the survey). This variable was created from the answer to the question ‘When was the last time you had vaginal intercourse?’.

Receiving contraceptive advice during or immediately after pregnancy is a binary variable (y/n) based on the answer to the question ‘Around the time of your last birth, did the health care that you received include information and advice about contraception?’. Satisfaction with contraceptive advice received is also a binary variable (Satisfied/Not Satisfied), created from the answer to the question ‘How satisfied were you with … advice about contraception to use after birth?’. Answers of ‘Very satisfied’ or ‘Satisfied’ were grouped to be ‘Satisfied’ and answers of ‘Neither satisfied or dissatisfied’, ‘Dissatisfied’ or ‘Very dissatisfied’ were grouped to be ‘Not satisfied’.

The precise wording of the survey questions and answer options used to create the variables in this analysis are provided in the Supporting Information (Data S1).

### 2.4 Statistical Analysis

First, we present descriptive statistics of the sample. Due to routing within the survey (and the non‐mandatory nature of the survey questions) the sample size varies between variables. Percentages presented throughout this paper are calculated based on the available data for each variable.

Logistic regression was used to estimate the odds of contraceptive initiation within two months and contraceptive use within one year of giving birth for each exposure variable, to produce unadjusted odds ratios (OR) and also age‐adjusted ORs. Following these initial analyses, multivariable analysis was used to estimate the effect of receipt of and satisfaction with contraceptive counselling on contraceptive initiation within two months and contraceptive use within one year, adjusting for all variables included in bivariate analysis (satisfaction with counselling was excluded from the multivariable analysis examining the effect of receiving contraceptive counselling). StataMP 18 was used for analysis.

### 2.5 Public Involvement

As part of the 2021 pilot survey [16], the LSHTM team worked with two panel groups, one with members aged 16–30 and one aged 31–50 for input into the survey design, content, and readability and acceptability of language used. For the 2023 survey, they worked with voluntary sector partners (Birth Companions, Brook, LGBT Foundation and Race Equality Foundation) on revisions to the survey content and to improve reach to groups identified as underserved in the pilot.

## 3 Results

### 3.1 Participants

Of the 59 332 people that took part in the 2023 RHS, 2503 participants (who had ever given birth) answered the question about whether they had given birth in the last year, among which 2073/2503 (82.8%) said they had. The mean age was 33 years, most participants were married (1401/2059, 68%), had a university degree (1708/2059, 83%) and were in paid employment (1433/2073, 69%). Most participants were White (1932/2060, 94%) and had been born in the UK (1840/2068, 89%). Almost half of participants were living in the two least deprived quintiles (869/1873, 46%). See Table 1 for detailed participant characteristics.

**TABLE 1:** Participant characteristics.

	Everyone who has given birth in last year *N* = 2073
Age (*N* = 2073)	
Range	17–50
Mean (SD)	33.1 (4.94)
Fertility intention (*N* = 2070)	
Do not want more children	545 (26.3%)
Not sure	711 (34.4%)
Definitely want, but not trying	752 (36.4%)
Currently trying	62 (3.0%)
Marital status (*N* = 2059)	
Not married	658 (32.0%)
Married	1401 (68.0%)
Education (*N* = 2059)	
No degree	351 (17.1%)
Degree	1708 (82.9%)
Employment (*N* = 2073)	
Not in paid employment	640 (30.9%)
In paid employment (including those on maternity leave)	1433 (69.1%)
Deprivation (IMD) (*N* = 1873)	
1 (most deprived)	207 (11.1%)
2	375 (20.0%)
3	422 (22.5%)
4	400 (21.4%)
5 (least deprived)	469 (25.0%)
Financial status (self‐reported) (*N* = 2068)	
Living comfortably	505 (24.4%)
Doing alright	911 (44.1%)
Just getting by	468 (22.4%)
Finding it quite difficult	143 (6.9%)
Finding it very difficult	41 (2.0%)
Ethnicity (*N* = 2060)	
White	1932 (93.8%)
Non‐White ethnic groups	128 (6.2%)
Religious (*N* = 2050)	
Not religious	1352 (65.9%)
Religious	698 (34.1%)
Migration (*N* = 2068)	
Born in UK	1840 (89.0%)
Born outside UK	228 (11.0%)
Parity (*N* = 2056)	
1	1153 (56.1%)
2	750 (365%)
3+	130 (7.4%)
Gender identity (*N* = 2073)	
Woman/girl	1056 (99.2%)
Other	17 (0.8%)
Sexual activity (*N* = 2066)	
Sex in last 6 months	1593 (77.1%)
No sex in last 6 months	473 (22.8%)
Received contraceptive advice during or immediately after pregnancy (*N* = 1999)	
No	514 (26.0%)
Yes	1485 (74.0%)
Satisfied with contraceptive advice received during or immediately after pregnancy (*N* = 1918)	
Not satisfied	874 (46.0%)
Satisfied	1044 (54.0%)

### 3.2 Contraceptive Initiation and Usage

Overall, 73% (1489/2044) of participants who had given birth in the last year reported that they were currently using contraception. However, only 6% (129/1988) reported initiating contraception immediately after birth, and the majority (1154/1998, 58%) had not initiated contraception within two months of giving birth.

### 3.3 Bivariate Analysis

#### 3.3.1 Contraceptive Initiation Within Two Months of Giving Birth

Following unadjusted bivariate analysis age, deprivation, parity, receiving contraception advice during pregnancy, and satisfaction with contraception advice received were all shown to be significantly associated with contraceptive initiation within two months of giving birth. Marital status, education, employment, financial status, ethnicity, religion and migration status were all found to be non‐significant. After adjusting for age, deprivation and parity became non‐significant (Table 2).

**TABLE 2:** Unadjusted and age‐adjusted bivariate analyses of factors associated with contraceptive initiation within two months of giving birth.

Variable	*N* (%)	Contraceptive initation within two months			Contraceptive initation within two months			% of participants who had iniated contraception within two months
		Unadjusted			Age‐adjusted			
		OR	95% CI	*p*	OR	95% CI	*p*	
Age								
< 25 as baseline	120 (5.8%)	1		< **0.001**	1		< **0.001**	54.20%
25–29	320 (15.4%)	0.79	0.51–1.20		0.79	0.51–1.20		48.20%
30–34	787 (38.0%)	0.73	0.49–1.07		0.73	00.49–1.07		46.30%
35–39	661 (31.9%)	0.49	0.33–0.73		0.49	0.33–0.73		36.80%
40+	185 (8.9%)	0.29	0.18–0.48		0.29	0.18–0.48		25.60%
Marital status								
Not married as baseline	658 (32.0%)	1		0.164	1		0.582	44.40%
Married	1401 (68.0%)	0.87	0.72–1.06		0.95	0.77–1.16		41.10%
Education								
No degree as baseline	351 (17.1%)	1		0.071	1		0.864	46.60%
Degree	1708 (82.9%)	0.81	0.64–1.01		0.98	076–1.27		41.30%
Employment								
Not in paid employment as baseline	640 (30.9%)	1		0.181	1		0.36	44.50%
In paid employment	1433 (69.1%)	0.88	0.72–1.06		0.91	0.75–1.11		41.30%
Deprivation (IMD)								
1 (most deprived) as baseline	207 (11.1%)	1		**0.022**	1		0.097	50.80%
2	375 (20.0%)	0.69	0.49–0.98		0.74	0.52–1.06		41.60%
3	422 (22.5%)	0.62	0.44–0.87		0.68	0.68–0.96		39%
4	400 (21.4%)	0.83	0.59–1.16		0.89	063–1.26		46.00%
5 (least deprived)	469 (25.0%)	0.63	0.45–0.88		0.7	0.50–0.98		39.40%
Financial status (self‐reported)								
Living comfortably as baseline	505 (24.4%)	1		0.704	1		0.842	41%
Doing alright	911 (44.1%)	1.05	0.84–1.32		1.01	0.80–1.27		42.20%
Just getting by	468 (22.4%)	112	0.87–1.45		0.98	0.75–1.28		43.80%
Finding it quite difficult	143 (6.9%)	1.13	0.77–1.65		0.93	0.63–1.39		43.90%
Finding it very difficult	41 (2.0%)	0.72	0.36–1.43		0.67	0.33–1.37		33.30%
Ethnicity								
White as baseline	1932 (93.8%)	1		0.328	1		0.31	42.60%
Non‐White ethnic groups	128 (6.2%)	0.83	0.57–1.21		0.82	0.56–1.20		38.00%
Religious								
Not religious as baseline	1352 (65.9%)	1		0.543	1		0.292	41.80%
Religious	698 (34.1%)	1.06	0.8–1.23		1.11	0.92–1.34		43.20%
Migration								
Born in UK as baseline	1840 (89.0%)	1		0.849	1		0.519	42.30%
Born outside UK	228 (11.0%)	0.97	0.73–1.29		1.1	0.82–1.47		41.60%
Parity								
1 as baseline	1153 (56.1%)	1		**0.012**	1		0.132	45.10%
2	750 (365%)	0.75	0.62–0.91		0.86	0.71–1.05		38.10%
3+	130 (7.4%)	0.94	0.66–1.33		1.19	0.83–1.71		43.50%
Received contraceptive advice during or immediately after pregnancy								
No as baseline	514 (26.0%)	1		**0.014**	1		**0.002**	37.60%
Yes	1485 (74.0%)	1.3	1.05–1.59		1,38	1.12–1.71		43.90%
Satisfied with contraceptive advice received during or immediately after pregnancy								
Not satisfied as baseline	874 (46.0%)	1		< **0.001**	1		< **0.001**	37.10%
Satisfied	1044 (54.0%)	1.59	1.32–1.91		1.68	1.40–2.03		48.40%

Participants over the age of 40 had the lowest odds of initiating contraception within two months of giving birth (OR = 0.29, 95% CI = 0.18 to 0.48, *p* < 0.001). Participants who had received contraception advice during or immediately after pregnancy had higher odds of initiating contraception within two months of giving birth, compared to those who had not received advice (OR = 1.38, 95% CI = 1.12 to 1.71, *p* = 0.002) and participants who were satisfied with the contraception advice they received had higher odds of initiating contraception within 2 months of giving birth (OR = 1.68, 95% CI = 1.40 to 2.03, *p* < 0.001).

#### 3.3.2 Contraceptive Use Within One Year of Giving Birth

After unadjusted bivariate analysis fertility intentions, age and sexual activity were the only variables shown to be significantly associated with contraceptive use within one year of giving birth. However, after adjusting for age, receiving contraceptive advice during pregnancy became significantly associated with contraceptive use (Table 3).

**TABLE 3:** Unadjusted and age‐adjusted bivariate analyses of factors associated with contraception use within one year of giving birth.

Variable	*N* (%)	Contraceptive use within one year			Contraceptive use within one year			% of participants who were using contraception within one year
		Unadjusted			Age‐adjusted			
		OR	95% CI	*p*	OR	95% CI	*p*	
Age								
< 25 as baseline	120 (5.8%)	1		< **0.001**	1		< **0.001**	78.50%
25–29	320 (15.4%)	1.05	0.62–1.76		1.05	0.62–1.76		79.30%
30–34	787 (38.0%)	0.84	0.52–1.34		0.84	0.51–1.34		75.30%
35–39	661 (31.9%)	0.66	0.41–1.07		0.66	0.41–1.07		70.70%
40+	185 (8.9%)	0.33	0.20–0.57		0.33	0.20–0.57		54.80%
Fertility intention								
Do not want more children as baseline	545 (26.3%)	1		< **0.001**	1		< **0.001**	78.10%
Not sure	711 (34.4%)	0.75	0.58–0.98		0.66	0.50–0.86		72.80%
Definitely want, but not trying	752 (36.4%)	0.8	0.62–1.04		0.63	0.47–0.83		74.10%
Currently trying	62 (3.0%)	0	0.02–0.10		0.04	0.02–0.08		14.80%
Marital Status								
Not married as baseline	658 (32.0%)	1		0.441	1		0.147	7.80%
Married	1401 (68.0%)	1.09	0.88–1.34		1.18	0.94–1.46		73.40%
Education								
No degree as baseline	351 (17.1%)	1		0.692	1		0.319	73.60%
Degree	1708 (82.9%)	0.95	0.73–1.23		1.16	0.87–1.54		72.60%
Employment								
Not in paid employment as baseline	640 (30.9%)	1		0.671	1		0.512	72.20%
In paid employment	1433 (69.1%)	1.05	0.85–1.29		1.08	0.87–1.34		73.10%
Deprivation (IMD)								
1 (most deprived) as baseline	207 (11.1%)	1		0.61	1		0.784	76.20%
2	375 (20.0%)	0.82	0.55–1.21		0.85	0.57–1.27		72.40%
3	422 (22.5%)	0.83	0.57–1.23		0.88	0.59 1.31		72.80%
4	400 (21.4%)	0.9	0.61–1.34		0.97	0.65–1.44		74.30%
5 (least deprived)	469 (25.0%)	0.75	0.52–1.10		0.82	0.56–1.21		70.70%
Financial status (self‐reported)								
Living comfortably as baseline	505 (24.4%)	1		0.503	1		0.501	71.40%
Doing alright	911 (44.1%)	0.9	0.71–1.15		0.87	0.68–1.11		70.50%
Just getting by	468 (22.4%)	1.12	0.84–1.50		0.99	0.74–1.34		75.00%
Finding it quite difficult	143 (6.9%)	0.86	0.57–1.31		0.74	0.48–1.15		75.70%
Finding it very difficult	41 (2.0%)	1.08	0.52–2.28		1.14	0.53–2.45		73.40%
Ethnicity								
White as baseline	1932 (93.8%)	1		0.511	1		0.464	73.10%
Non‐White ethnic groups	128 (6.2%)	0.88	0.59–1.30		0.86	0.58–1.29		70.40%
Religious								
Not religious as baseline	1352 (65.9%)	1		0.996	1		0.619	72.80%
Religious	698 (34.1%)	1.00	0.81–1.23		1.05	0.86–1.30		72.80%
Migration								
Born in UK as baseline	1840 (89.0%)	1		0.707	1		0.728	73.10%
Born outside UK	228 (11.0%)	0.94	0.69–1.28		1.06	0.77–1.45		71.90%
Parity								
1 as baseline	1153 (56.1%)	1		0.821	1		0.071	72.60%
2	750 (365%)	1.03	0.83–1.26		1.2	0.96–1.49		73.10%
3+	130 (7.4%)	1.13	0.77–1.67		1.51	1.01–2.27		75.00%
Sexual activity								
Sex in last 6 months as baseline	1593 (77.1%)	1		< **0.001**	1		**0.003**	80.10%
No sex in last 6 months	473 (22.8%)	0.23	0.18–0.29		0.25	0.20–0.31		48.20%
Received contraceptive advice during or immediately after pregnancy								
No as baseline	514 (26.0%)	1		0.102	1		**0.027**	70.40%
Yes	1485 (74.0%)	1.21	0.96–1.51		1.29	1.03–1.63		74.20%
Satisfied with contraceptive advice received during or immediately after pregnancy								
Not satisfied as baseline	874 (46.0%)	1		0.481	1		0.242	73.60%
Satisfied	1044 (54.0%)	1.08	0.88–1.32		1.13	0.92–1.40		75.00%

*Note*: Bold values indicate p‐values are statistically significant (*p* ‐value of 0.05 or less).

Participants over the age of 40 had the lowest odds of using contraception within one year of giving birth, compared to those under 25 years (OR = 0.33, 95% CI = 0.20 to 0.57, *p* < 0.001). Participants currently trying had the lowest odds of using contraception within one year (OR = 0.04, 95% CI = 0.02 to 0.08, *p* < 0.001) compared to those who didn't want a pregnancy. Participants who had received contraception advice during or immediately after pregnancy had greater odds of using contraception within one year of giving birth, compared to those who hadn't received advice (OR = 1.29, 95% CI = 1.03 to 1.63, *p* = 0.027).

### 3.4 Multivariable Analysis

When all variables (apart from satisfaction with contraceptive advice) were included in the multivariable analysis, receiving contraceptive advice during or immediately after pregnancy remained significantly associated with contraceptive initiation within two months of giving birth and contraceptive use within one year of giving birth, indicating that the effect is independent of the other variables. Participants who received contraceptive advice had greater odds of initiating contraception within two months (OR = 1.52, 95% CI = 1.20–1.92, *p* < 0.001) and one year of giving birth (OR = 1.39, 95% CI = 1.08–1.78, *p* = 0.01). Satisfaction with advice received also remained significantly associated with contraceptive initiation within two months of giving birth; participants who were satisfied with the contraception advice they received had greater odds of initiating contraception within two months of giving birth compared to those who were not satisfied (OR = 1.70, 95% CI = 1.36–2.12, *p* < 0.001). See Table S1 for multivariable table.

### 3.5 Contraceptive Effectiveness

The most common methods of contraception used in the year following birth were least effective methods (998/2044, 49%), followed by no method of contraception (555/2044, 27%), effective methods (249/2044, 12%) and then most effective methods (242/2044, 12%). Within all fertility intention groups (apart from those currently trying to get pregnant), less effective methods were the most used methods, followed by no method of contraception. (See Table 4).

**TABLE 4:** Breakdown of contraceptive effectiveness used within 1 year of giving birth.

	Total sample *N* = 2044		Do not want *N* = 538		Not sure *N* = 698		Want—not trying *N* = 744		Currently trying *N* = 61	
	*N* (%)	95% CI	*N* (%)	95% CI	*N* (%)	95% CI	*N* (%)	95% CI	*N* (%)	95% CI
No Method	555 (27.2%)	25.3–29.1	118 (21.9%)	18.6–25.6	190 (27.2%)	24.0–30.6	193 (25.9%)	22.9–29.2	52 (85.2%)	74.0–92.1
Less effective methods (condoms, fertility awareness, withdrawal)	998 (48.8%)	46.7–51.0	252 (46.8%)	42.7–51.1	355 (50.8%)	47.2–54.6	383 (51.5%)	47.9–55.1	7 (11%)	5.5–22.2
Effective methods (contracetive pill, injection, patch)	249 (12.2%)	10.8–13.7	55 (10.2%)	7.9–13.1	83 (11.8%)	9.7–14.5	109 (14.6%)	12.3–17.4	2 (3%)	0.8–12.2
Most effective methods (IUD, implant, sterilisation)	242 (11.8%)	10.5–13.3	113 (21.0%)	17.8–24.7	70 (10.0%)	8.0–12.5	59 (7.9%)	6.2–10.1	0 (0%)	—

Use of the most effective contraception methods was less common among older women (OR = 0.24, 95% CI = 0.15–0.38, *p* < 0.001), those with a degree (OR = 0.72, 95% CI = 0.58–0.890, *p* = 0.003), women who were not sexually active (OR = 0.27, 95% CI = 0.22–0.37, *p* < 0.001) and women who were currently trying to get pregnant (OR = 0.04, 95% CI = 0.02–0.08, *p* < 0.001). In contrast, individuals struggling financially (OR = 1.61, 95% CI = 0.86–3.02, *p* = 0.017), those with three or more children (OR = 1.6, 95% CI = 1.16–2.28, *p* = 0.02), and those satisfied with contraceptive advice received during or immediately after pregnancy were more likely to be using the most effective contraception methods (OR = 1.39, 95% CI = 1.17–1.64, *p* < 0.001). See Table S2 for detailed analysis table.

## 4 Discussion

### 4.1 Main Findings

Our results show that over half of participants had not initiated contraception within two months of giving birth. Satisfaction with contraceptive advice received was strongly associated with timely initiation of contraception within the postnatal period. Furthermore, receiving advice was significantly associated with contraceptive use within one year of giving birth. These findings highlight the antenatal period as an important window of opportunity to discuss and advise women and birthing people about their post‐birth contraceptive choices.

Encouragingly, most participants received contraceptive advice during pregnancy; however, only 54% were satisfied with it. While the specific content of counselling was not captured in this survey, the FSRH recommends that antenatal counselling cover available methods, effectiveness, individual considerations, fertility intentions, and optimal interpregnancy intervals [2]. Satisfaction with counselling has been linked to knowledgeable, open‐minded providers and patient‐centred support, though it remains a complex concept [18]. Further qualitative research is needed to better understand the factors that shape satisfaction, particularly given our findings suggest that contraceptive counselling can impact postnatal contraceptive use.

Despite the overwhelming majority of participants not currently trying for further pregnancies, usage of effective methods of contraception in the year after giving birth is limited. Least effective methods (e.g., condoms, fertility awareness, lactational amenorrhea, withdrawal), which are associated with higher failure rates, were the most commonly used methods of contraception within our sample. Our study indicates that older women are less likely to be using the most effective methods of contraception, while women with more children or those who were satisfied with the contraception advice they received during or immediately after pregnancy were more likely to be using the most effective methods. When practiced correctly, lactational amenorrhea can be an effective contraceptive method; however, its effectiveness decreases if certain conditions are not met—specifically, exclusive breastfeeding, absence of menstruation, and the baby being six months old or younger. Since this evaluation assesses contraception within one year of giving birth, we classified lactational amenorrhea as less effective.

### 4.2 Strengths and Limitations

A key strength of this study is that it is the first national study of contraception after birth, and the first to map out usage of contraception and method mix against current fertility intentions. A large sample was achieved, at a relatively low cost, with good regional representation [16]. There was a very low non‐response rate for all items. The survey collected data on a combination of variables which allows an examination of the association between a broad range of factors and both contraception initiation and use in the post‐partum period. Furthermore, the questions were asked in a separate context to where the contraceptive advice was delivered, potentially reducing response bias.

In this analysis, we included participants who reported no sexual activity in the last six months. Without information on the reasons for sexual inactivity, contraceptive non‐use and the timing of birth, we cannot reliably identify individuals who are at risk of pregnancy. Excluding these participants would require assumptions about their sexual inactivity and contraceptive use, which could bias the results. As a result, we have not been able to fully describe and understand unmet need for contraception within our population.

There is also the potential for recall bias, which may affect our results, for example individuals who initiate or use contraception are potentially more likely to remember receiving (or report higher satisfaction with) contraceptive counselling during pregnancy. Finally, this was a convenience sample (with participants being recruited through social media and organisation networks), and participants from the most deprived quintile, those without a degree and minority ethnic groups were underrepresented within our sample, therefore our results are not generalisable to the whole postnatal population of England. Ensuring the inclusion of these underserved groups in future research is crucial to accurately reflect their experiences and needs.

### 4.3 Interpretation

There was a strong correlation between fertility intentions and contraceptive use within one year of giving birth, which is consistent with our expectations and the wider literature [19]. This highlights the importance of discussing fertility intentions as part of contraceptive counselling. Tools such as the Desire to Avoid Pregnancy scale, which has been validated for use in the UK [20, 21], could be used to identify an individual's fertility intentions as part of contraceptive counselling.

Older participants were the least likely to be using contraception at both two months and one year post birth. This aligns with Moffat's findings [8] on demographic predictors of post birth contraception initiation in the North East of England, which showed that younger women and birthing people were significantly more likely to access medically prescribed contraception with the first 8 weeks post birth. Our results also reflect national data from England and Wales showing a rise in abortions among over 35 year olds [7]. Together, these findings suggest an increased unmet need for contraception among older women and birthing people. The limited usage of effective contraception in our study's population of participants who had recently given birth adds weight to the case for better information and access to methods in the postpartum period. Access to long‐acting reversible contraception (LARC) in the community is known to be becoming scarcer [22] and the additional pressures of having a baby add another potential barrier to access [23]. There is growing evidence to suggest that initiation of LARC or the provision of short acting reversible methods prior to discharge from maternity services is feasible, safe, and wanted by women and birthing people [6, 24].

Finally, there was a small proportion of survey participants (3%) who were actively trying to conceive within one year of giving birth. It is advised that women and birthing people avoid pregnancy for at least 18 months after giving birth to prevent short interpregnancy interval complications. While NICE guidance does suggest that advice around interpregnancy intervals should take into account individual circumstances, such as advising shorter intervals for older women or birthing people concerned about fertility [25]. Within our study, the average age of the participants who were currently trying to get pregnant did not differ significantly from the whole sample, therefore age does not seem to be an explanatory factor. This study's results therefore indicate that, for some individuals, having children close together may be desirable. Further investigation is needed to understand the motivation behind short interpregnancy intervals and counselling about interpregnancy intervals should take this into consideration.

## 5 Conclusion

Overall, this study highlights an association between receipt of, and satisfaction with, contraceptive counselling and postnatal contraceptive initiation and use, indicating that comprehensive contraceptive counselling should be a standard part of maternity care. Further research is needed to better understand what contributes to satisfactory counselling experiences. Any future work must include underserved groups to ensure equitable access to postnatal contraception for all women and birthing people.

## Author Contributions

R.S.F., M.J.P., and O.M. conducted the 2023 Reproductive Health Survey. C.S., N.P., and A.H. conceptualised and designed this secondary analysis, with input from M.J.P., O.M., and R.S.F. C.S. performed the analyses with input from all authors. C.S. and A.H. drafted the initial manuscript; C.S. then prepared all subsequent revisions. All authors reviewed and commented on all revisions and approved the final version for journal submission.

## Ethics Statement

Ethical approval for the Reproductive Health Survey was granted by the LSHTM Observational/Intervention Research Ethics Committee (LSHTM Ethics ref: 29389). Further approval was not required from UCL as the work involved a secondary data analysis in collaboration with the LSHTM team.

## Conflicts of Interest

The authors declare no conflicts of interest.

## Supporting information


Data S1.

**Table S1.** Multivariate analysis.

**Table S2.** Bivariate analysis.

## Data Availability

The data that support the findings of this study are available from the Department of Health and Social Care. Restrictions apply to the availability of these data, which were used under licence for this study. Data are available from the author(s) with the permission of the Department of Health and Social Care.
