# Supplementary material for: Factors Associated With Contraceptive Initiation and Use Among Women Who Have Given Birth in the Last Year: Findings From the 2023 Women's Reproductive Health Survey
Source: BJOG. 2025 Jul 9;132(11):1615–25. doi: 10.1111/1471-0528.18264 (PMC12411649; doi:10.1111/1471-0528.18264)
Supplement: Supplementary file 1 — Data S1. [file BJO-132-1615-s001.docx]

**Supplementary Materials: Factors associated with contraceptive initiation and use amongst women who have given birth in the last year: findings from the 2023 Women’s Reproductive Health Survey.**

**Authors:** Catherine Stewart^1^, Amy Hough^2,3^, Melissa J Palmer^4^, Ona McCarthy^4^, Rebecca S French^4^, Neha Pathak^2,5^.

**Affiliations**:

^1^ Reproductive Health, UCL EGA Institute of Women’s Health.

^2^ Institute of Global Health, University College London

^3^ University Hospitals Birmingham NHS Trust

^4^ Faculty of Public Health and Policy, The London School of Hygiene & Tropical Medicine

^5^ Guy’s & St Thomas’s NHS Trust.

Corresponding Author: Catherine Stewart, 74 Huntley Street London WC1E 6DE, [catherine.stewart.20@ucl.ac.uk](mailto:catherine.stewart.20@ucl.ac.uk)

Contents

[Supplementary Materials 1: Wording of Questions and Answer Options 2](#_Toc199323996)

[Table S1: Multivariable Analysis – contraceptive use within two months and use within one year. 7](#_Toc199323997)

[Table S2: Bivariate Analysis – contraceptive effectiveness 8](#_Toc199323998)

# Supplementary Materials 1: Wording of Questions and Answer Options

Have you ever been pregnant, even if you did not carry the baby to term?

- Yes
- No

Have you ever given birth?

- Yes
- No

How many times have you had a pregnancy that ended in the birth of one or more live babies?

Have you given birth to a live baby in the last 12 months?

- Yes
- No

The next question is about having more children. Which of these statements best describes the way you feel about having more children? Please include different ways of having children, including adoption and having IVF.

- I would definitely like more children and I’m currently trying
- I would definitely like more children but I’m not currently trying
- I might like more children in the future - I’m not sure yet
- I would definitely not like more children
- I don't know

Around the time of your last birth, did the health care that you received include information and advice about contraception?

- Yes
- No
- Unsure

How satisfied were you with the following services relating to your pregnancy and birth?

Advice about contraception to use after birth

- Very satisfied
- Satisfied
- Neither satisfied or dissatisfied
- Dissatisfied
- Very dissatisfied
- Not applicable

Did you start using a method of contraception within two months of this pregnancy ending?

- Yes, immediately after delivering my baby
- Yes, following my 6-8 week postnatal check with my GP
- Yes, at another time within two months of the pregnancy ending
- No

When was the last time you had vaginal intercourse?

*In this survey because of the focus on reproductive health, vaginal intercourse is defined as a penis inside a vagina. Your answers to this question help us understand the next set of questions about contraception and pregnancy.*

- Never had vaginal intercourse
- In the last 7 days
- Between 7 days and 4 weeks ago
- Between 4 weeks and 6 months ago
- Between 6 months and 1 year ago
- Between 1 and 5 years ago
- Longer than 5 years ago
- Prefer not to say

What contraceptive method are you currently using? This could be a method to prevent pregnancy or sexually transmitted infections, or for any other reason (e.g. pill use to help with acne or periods).

Please tick main method

- No method used at the moment
- Male condom
- Female condom
- Cap/diaphragm
- Partner has been sterilised (had a vasectomy)
- I have been sterilised (tubal ligation, 'tubes tied')
- Combined oral contraceptive pill (e.g. microgynon, marvelon, yasmin)
- Progesterone only contraceptive pill (e.g. cerazette, cerelle)
- Contraceptive pill - don't know which type
- Hormonal IUS (e.g., Mirena, Jaydess, Kyleena, Levosert)
- Vaginal ring (e.g. NuvaRing)
- Contraceptive patch (e.g. EVRA)
- Injections
- Implant
- Emergency contraceptive pill/morning after pill
- Emergency copper coil/intra-uterine device (IUD)
- Safe period/ calendar method/ rhythm method
- Withdrawal (not ejaculating in a partner's vagina)
- Fertility awareness apps or devices
- Avoiding penetrative sex
- Copper coil/intra-uterine device (IUD)
- Spermicides (foams/gels/sprays/pessaries)
- Lactational amenorrhoea method (LAM)
- Other method of contraception

What is your postcode?

*We will not use your postcode to identify you. It will be used to understand health and services in different areas in England.*

What is you age?

What is your legal marital or registered civil partnership status?

- Never married and never registered a civil partnership
- Married / in a civil partnership
- Divorced / formerly in a civil partnership that is now legally dissolved
- Widowed / surviving partner from a registered civil partnership

What is your ethnic group?

- White: English, Welsh, Scottish, Northern Irish or British
- White: Irish
- White: Gypsy or Irish Traveller
- Any other White background
- Mixed or Multiple ethnic groups: White and Black Caribbean
- Mixed or Multiple ethnic groups: White and Black African
- Mixed or Multiple ethnic groups: White and Asian
- Any other Mixed or Multiple ethnic background
- Asian or Asian British: Indian
- Asian or Asian British: Pakistani
- Asian or Asian British: Bangladeshi
- Asian or Asian British: Chinese
- Any other Asian background
- Black, Black British, Caribbean or African: African
- Black, Black British, Caribbean or African: Caribbean
- Any other Black, Black British, Caribbean or African background
- Other ethnic group: Arab
- Any other ethnic group

Were you born in...?

- England
- Wales
- Scotland
- Northern Ireland
- Another country

What is your religion?

- No religion
- Christian (including Church of England, Catholic, Protestant and all other Christian
- denominations)
- Buddhist
- Hindu
- Jewish
- Muslim
- Sikh
- Any other religion

Have you got a degree or similar qualification?

*For example, university degree, foundation degree, HND or HNC NVQ level 4 and above,*

*teaching or nursing*

- Yes
- No

Which of these descriptions applies to what you were doing last week, that is, in the seven days ending last Sunday?

*Tick all that apply*

- Going to school, college or university full-time (including on vacation)
- In paid employment or self employed (or temporarily away)
- On a Government scheme for employment training
- Doing unpaid work for a business that you own, or that a relative owns
- Waiting to take up paid work already obtained
- Looking for paid work or a Government training scheme
- Intending to look for work but prevented by temporary sickness or injury (if more than 28
- days, select 'permanently unable to work' below.)
- Permanently unable to work because of long-term sickness or disability
- Retired from paid work
- Looking after home or family
- Doing something else

How well would you say you yourself are managing financially these days?

- Living comfortably
- Doing alright
- Just getting by
- Finding it quite difficult
- Finding it very difficult

# Table S1: Multivariable Analysis – contraceptive use within two months and use within one year.

Table S1: Multivariable analysis investigating the effect of receiving contraceptive advice during or immediately after pregnancy and satisfaction with contraceptive advice received on contraceptive initiation within two months and contraceptive use within one year. Adjusted for age, marital status, education, employment, deprivation, financial status, ethnicity, religion, migration and parity.

# Table S2: Bivariate Analysis – contraceptive effectiveness

Table S2: Bivariate analysis investigating the factors associated with contraceptive effectiveness. (Contraceptive effectiveness is a categorical variable – no method, least effective methods (condoms, fertility awareness, withdrawal, lactational amenorrhea), effective methods(contraceptive pill, injection, patch), most effective methods(IUD, implant, sterilisation).)
